# Gamma spectrometry in the ITWG CMX-4 exercise

**DOI:** 10.1007/s10967-017-5667-2

**Published:** 2018-01-05

**Authors:** L. Lakosi, J. Zsigrai, A. Kocsonya, T. C. Nguyen, H. Ramebäck, T. Parsons-Moss, N. Gharibyan, K. Moody

**Affiliations:** 1 Nuclear Security Department, Hungarian Academy of Sciences, Centre for Energy Research, 29-33 Konkoly-Thege M., Budapest, 1121 Hungary; 2 European Commission, Joint Research Centre, Directorate for Nuclear Safety and Security, P.O.Box 2340, 76125 Karlsruhe, Germany; 3 Swedish Defence Research Agency, CBRN Defence and Security, Cementvägen 20, SE-901 82 Umeå, Sweden; 4 Department of Chemistry and Chemical Engineering, Chalmers University of Technology, Kemivägen 4, SE-412 58 Göteborg, Sweden; 5 Lawrence Livermore National Laboratory, P.O. Box 808, L-186, Livermore, CA 94551 USA

**Keywords:** Intercomparison, Nuclear forensics, Nondestructive assay, Gamma spectrometry, Uranium isotopic composition, Radiochronometry

## Abstract

Low enriched uranium samples of unknown origin were analyzed by 16 laboratories in the context of a Collaborative Materials Exercise (CMX), organized by the Nuclear Forensics International Technical Working Group (ITWG). The purpose was to compare and prioritize nuclear forensic methods and techniques, and to evaluate attribution capabilities among participants. This paper gives a snapshot of the gamma spectrometric capabilities of the participating laboratories and summarizes the results achieved by gamma spectrometry.

## Introduction

This paper presents the state of practice in gamma spectrometry for nuclear forensics exercises. The Nuclear Forensics International Technical Working Group (ITWG) organized its fourth interlaboratory exercise in 2014, called Collaborative Materials Exercise (CMX-4) [1]. This paper documents the collective experience with gamma spectrometry during the CMX-4 exercise and it gives a snapshot of the applied approaches.

Nuclear forensics is the analysis of intercepted nuclear or other radioactive material to provide evidence for nuclear attribution in a legal context. The goal of the analysis is to identify the composition, origin, and intended use of interdicted nuclear or radiological samples, containers, and transport vehicles. Nuclear forensic analysis includes the characterization of the material and correlation with its production history [2]. The CMX-4 represents the second paired-comparison exercise organized to improve international cooperation and communication in case of a nuclear security event.

Three oxide samples of low enriched uranium (LEU) were selected as the materials to be characterized during the CMX-4 exercise. A scenario was included in which a seizure of nuclear material occurred and forensic analysis was requested. Laboratories were instructed to submit assessment reports in a 24 h, 1 week, and 2 month time frame. Participating laboratories categorized and characterized the exercise materials, and performed nuclear forensic evaluations. Each of the 16 participating laboratories was assigned a code name by the organizers to ensure anonymity and confidentiality of data.

Among the methods used in nuclear forensics, high-resolution gamma spectrometry (HRGS) is a relatively rapid nondestructive analytical technique. Advantages include preservation of evidence and no, or a minimal, need for sample preparation. A disadvantage is that it suffers from higher uncertainty compared to destructive techniques, such as mass spectrometry (MS) [3]. This paper presents the isotopic composition, age, and signatures of the neutron irradiation history of the three LEU samples determined by gamma spectrometry. These values are compared to the community average values determined by mass spectrometry.

## Sample description

Participants were provided three samples of similar size and mass: ES-1, ES-2, and ES-3. The ES-1 sample consisted of a physically homogenous, fine black U-oxide powder. ES-2 and ES-3 samples were dark gray, homogeneous, UO_2_ pellets with smooth surfaces. Representative physical sample properties are shown in Table 1. Pellets are made of UO_2_, whereas ES-1 was a mixture of UO_2_ and higher U-oxides. Further details on the exercise samples are provided in the introductory article of this Special Section [1].

**Table 1:** Average physical properties of the samples used in the exercise

Sample ID	Physical form	Mass, g ± 0.1	Size, mm ± 0.1		Approximate isotopic abundance, mass%		
			Diam.	Height	^234^U	^235^U	^238^U
ES-1	Powder	2.9			0.025	2.9	97.1
ES-2	Pellet	2.4	9.2	3.7	0.018	2.19	97.81
ES-3	Pellet	2.5	9.1	3.7	0.025	2.9	97.1

## Determining major U isotopic (^234^U, ^235^U, ^238^U) abundances

The samples were assayed first using HRGS for the 24 h and 1 week reports. Spectra were generally acquired by high purity germanium (HPGe) detectors for about 30–60 min in the case of the 24 h reports, whereas much longer measurement times were used for the 1 week reports.

Most participants of the exercise determined the major U isotopic (^234^U, ^235^U, and ^238^U) abundances using computer codes [4, 5] for automatic spectrum evaluation. These codes are based on so-called relative efficiency calibration (or intrinsic calibration) method [6]. The relative efficiency curve is obtained from the spectrum of the measured sample, thus the attenuation both in the sample (self-attenuation) and in absorbers (shielding) are taken into account. Therefore, the method does not depend on the sample size, geometry, physical, and chemical state. As all the information for determining the isotope ratios is present in the spectrum of the sample, no reference materials are required for calibration. However, for quality control purposes and for demonstrating laboratory performance, it is recommended to use a set reference materials.

Manual evaluation also occurred, after primary processing (measurement control, peak shape fitting, determining peak area, and deconvolution of overlapping peaks) by codes (FitzPeaks, PeakEasy, and Gammavision), followed by application of intrinsic self-calibration. For example, peaks of ^214^Bi (for age dating) and descendants of ^232^U were evaluated manually.

Table 2 summarizes the gamma spectrometers and software used for determining the isotopic composition of the samples in the CMX-4 exercise. Only participants sharing their results for this paper are listed. High-efficiency coaxial HPGe detectors used for age dating and identifying reprocessed uranium are not included here.

**Table 2:** HPGe detectors and software used by participants to determine the abundance of the major U isotopes

Lab code	Detector	Software
Michelangelo	ORTEC Micro-Detective-HX, coaxial, diam. 50 mm, height 30 mm, electrically cooled,	Identify FRAM 5.1, (In-house param. set: V_CX120-1010keV_microdetective)
Van Gogh	Canberra Falcon5000, broad-energy, diam. 61.8 mm, height 31.70 mm, electrically cooled	Manual, intrinsic efficiency calibration
Monet	Ortec GMX40P, coaxial, diam 63.0 mm, height 63.8 mm, electrically cooled	FRAM 5.2, (Param. set: ULEU-coax120-1010)
Rembrandt	Canberra GL0510, planar, diam. 24.8 mm, height 10 mm, active area 500 mm^2^	MGAU V.3.2
Renoir	Canberra GL0515R, planar diam. 25.2 mm, height 15 mm, active area 500 mm^2^	MGAU V.4.2
Picasso	For 24 h report: Canberra GL2020R, planar, diam. 50.5 mm, height 20 mm For 1 week report: ORTEC GLP10180/07P4, planar, diam. 10 mm, height 7 mm	MGAU V.4.2
Buonarroti	Canberra GL0210R, planar, diam. 16 mm, height 10 mm, active area 200 mm^2^	MGAU V.4.2
Pollock	Canberra BE3820, broad-energy Ge, diam. 70 mm, height 20 mm	U235 v1.51 (MGA ++)
Gauguin	Ortec, coaxial (averages from different detectors were used) GEM30P4-70: diam. 54.8 mm, height 51.0 mm GEM-20180-S: diam. 51.0 mm, height 50.7 mm GEM-10195: diam. 42.7 mm, height 49.0 mm GEM-13180: diam. 4.0 mm, height 50.1 mm	GAMANAL

For ^234^U most participants obtained results that correlate well with the average of mass spectrometric results for all samples (Table 3). Biases fall within expanded uncertainty (*k* = 2) limits, amounting to 10–20% relative uncertainty. Older versions of MGAU tend to underestimate ^234^U due to inaccuracies in the extrapolation of the intrinsic efficiency curve to 120.9 keV [7]. Evaluation by the GAMANAL code also resulted in unrealistically low ^234^U abundance.

**Table 3:** Available data of isotope abundances in mass %, measured by gamma spectrometry

Lab code/software	ES-1 powder			ES-2 pellet			ES-3 pellet		
	^234^U	^235^U	^238^U	^234^U	^235^U	^238^U	^234^U	^235^U	^238^U
Michelang./Identify FRAM	_	1.93 ± 0.14 (Identify) 3.03 ± 0.18 (FRAM)	_	_	1.50 ± 0.06 (Identify)2.14 ± 0.1 (FRAM)	_	_	1.93 ± 0.32 (Identify) 3.41 ± 0.41 (FRAM)	_
Van Gogh/manual	_	3.3 ± 0.5	_	_	2.6 ± 0.2	_	_	2.6 ± 0.5	_
Monet/(FRAM)	0.023 ± 0.004	2.614 ± 0.140	97.34 ± 0.140	0.019 ± 0.006	2.290 ± 0.138	97.677 ± 0.140	0.016 ± 0.012	2.659 ± 0.32	97.308 ± 0.322
Rembrandt/MGAU V3.2	0.028 ± 0.016	2.85 ± 0.16	97.12 ± 0.18	0.014 ± 0.012	2.11 ± 0.15	97.86 ± 0.15	0.016 ± 0.012	2.72 ± 0.15	97.25 ± 0.16
Renoir/MGAU V4.2	0.023 ± 0.0024	2.868 ± 0.047	97.11 ± 0.0475	0.0154 ± 0.0032	2.202 ± 0.0424	97.78 ± 0.0430	0.0244 ± 0.0041	2.833 ± 0.059	97.143 ± 0.06
Picasso/MGAU V4.2	0.026 ± 0.004	2.823 ± 0.039	97.151 ± 0.040	0.018 ± 0.004	2.138 ± 0.034	97.844 ± 0.035	0.025 ± 0.004	2.787 ± 0.041	97.188 ± 0.041
Buonarroti/MGAU V4.2	0.025 ± 0.0014	2.851 ± 0.038	97.124 ± 0.040	0.0192 ± 0.0012	2.154 ± 0.030	97.827 ± 0.030	0.0250v± 0.0010	2.861 ± 0.036	97.114 ± 0.036
Pollock/U235 V1.51	0.025 ± 0.010	2.91 ± 0.020	97.07 ± 0.020	0.019 ± 0.010	2.16 ± 0.020	97.83 ± 0.020	0.019 ± 0.010	2.86 ± 0.020	97.12 ± 0.020
Gauguin/GAMANAL	0.01565 ± 0.00072	2.752 ± 0.080	97.233 ± 0.080	0.00747 ± 0.00075	2.21 ± 0.12	97.78 ± 0.12	0.00916 ± 0.00092	2.99 ± 0.15	97.00 ± 0.15
Community average MS without outliers	0.02433 ± 0.00093	2.875 ± 0.040	97.076 ± 0.0082	0.01881 ± 0.00108	2.190 ± 0.040	97.789 ± 0.046	0.02441 ± 0.0083	2.875 ± 0.043	97.084 ± 0.084

Expanded uncertainties’ coverage factor *k* = 2. The community average of mass spectrometric results is calculated without the outliers, and its expanded standard deviation (*k* = 2) is given

Concerning ^235^U, all participants recognized the LEU character of the samples, regardless of the detector-type and software used. Participants found similar enrichment values of around 2.6–2.9% for samples ES-1 and ES-3, and 2.1–2.3% for the sample ES-2 (Table 3). The majority of values reported by participants provided a means to differentiate ES-1 and ES-3 from ES-2, regardless of the method used.

MGAU v4.2 results from spectra taken by a planar detector compared very well to the average ^235^U enrichment measured by mass spectrometry for all three samples. The slight underestimation of the ^235^U content by MGAU may come from the coincidence summing losses in the peaks of ^235^U [8]. Some evaluations of spectra taken by coaxial and broad-energy detectors resulted in significantly higher ^235^U content. This could be due to coincidence summing losses in the high-energy peaks from ^234m^Pa, which leads to an underestimation of the activity ratio ^238^U(^234m^Pa)/^235^U and therefore an overestimation of ^235^U [9]. The ^235^U enrichment estimates by the Identify software were significantly lower than values reported by mass spectrometry. One reason for the discrepancy could be wrong assumptions on the sample matrix, similar to what previously was observed for low resolution measurements [10].

Regarding ^238^U, mass abundances of 97.2 ± 0.1% were reported for ES-1 and ES-3, whereas 97.8 ± 0.1% for ES-2, in agreement with mass spectrometric results.

It can be concluded that the inventory of the three major uranium isotopes established by HRGS generally agreed with mass spectrometric results within expanded uncertainties and confirmed the LEU character of the samples. The accuracy was generally enough for distinguishing ES-1 and ES-3 from ES-2. Exceptions for ^235^U results came from some participants using coaxial germanium detector. Discrepancies for ^234^U results took place mainly for participants using outdated computer routines [7], or efficiency transfer algorithms based on point-source efficiency calibrations instead of relative, intrinsic efficiency calibrations.

**Fig. 1:**
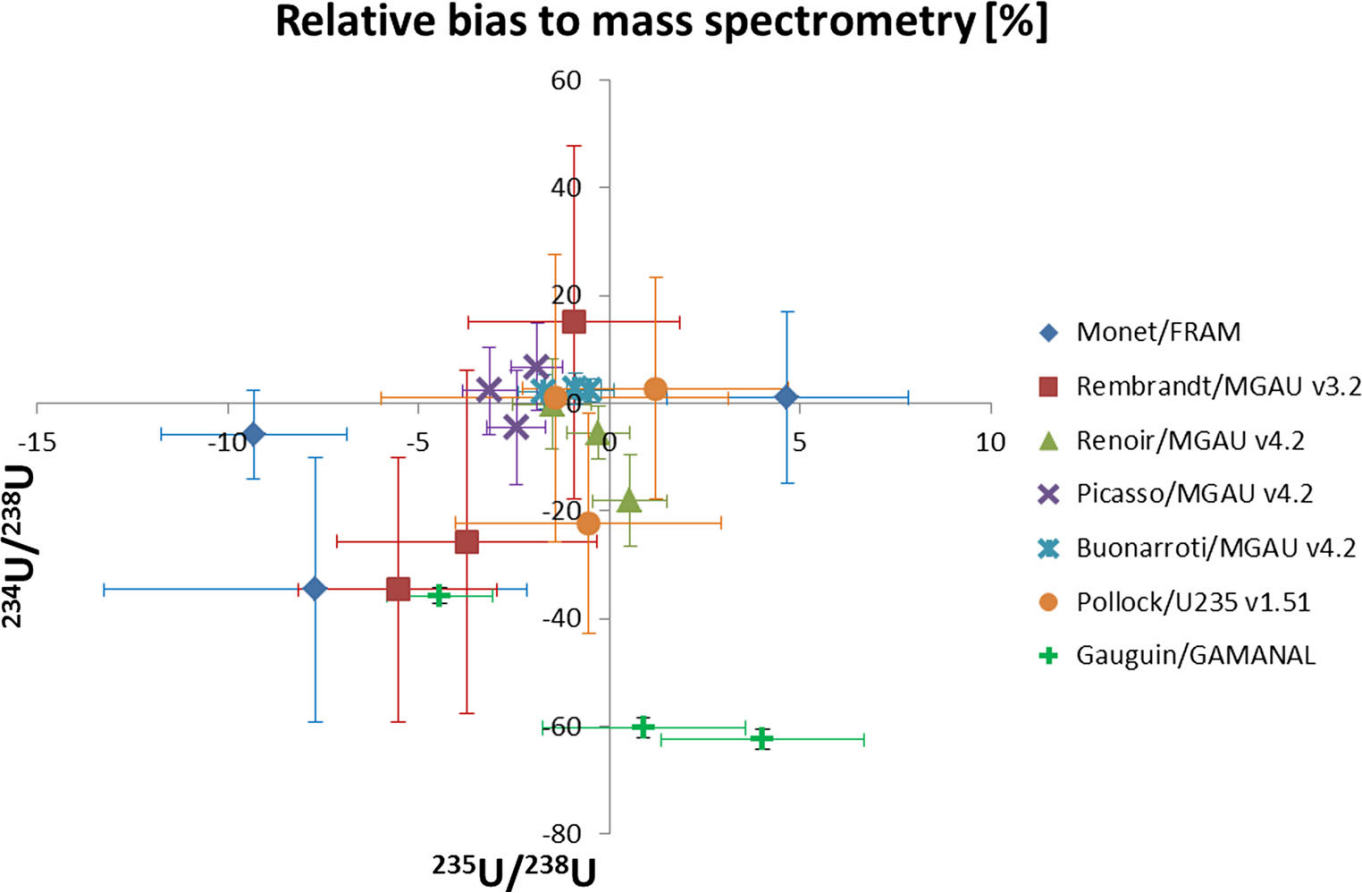
Relative biases with respect to the community average mass spectrometric result. The bias of the mass ratio ^234^U/^238^U versus the bias of ^235^U/^238^ is shown for all three samples. Uncertainties are displayed with a coverage factor of *k* = 1

Results of uranium isotopic abundance measurements by gamma spectrometry and community average values by mass spectrometry for comparison are summarized in Table 3. The mass spectrometry average is calculated from the data given in graphical form in the CMX-4 After-Action report [1]. For this calculation the outliers were removed. A three-isotope plot of the relative biases with respect to mass spectrometry for the ^234^U/^238^U and ^235^U/^238^ isotopic ratios is shown Fig. 1.

## U age dating

The model age (time elapsed since the last chemical purification) of the material is important for determining of the origin of nuclear material outside of regulatory control. The daughter/parent ratio as a function of decay time is widely used for determining the age of radioactive samples [11, 12]. Gamma spectrometric uranium age dating is nondestructive and suitable for relatively rapid assay. The method does not require the use of reference materials of known ages, nor radionuclide standards for method calibration. Usually there is no need to take subsamples or dismantle the investigated items, so preservation of evidence can easily be ensured. The method works particularly well for high-enriched and aged material. Its limits appear for low-enriched material and in sensitivity to background.

Uranium age dating by HRGS is based on the ^234^U → ^230^Th → ^226^Ra chronometer [13–19]. ^234^U can be detected by its 120.9 keV gamma line, whereas ^230^Th has no measurable gamma rays. The next member of the ^234^U decay series is ^226^Ra, of which the only gamma-line at 186.2 keV overlaps with 185.7 keV line of ^235^U. However, its short lived descendants ^214^Pb and ^214^Bi have measureable gamma lines.

The time needed for secular equilibrium between ^226^Ra and ^214^Bi is about 3 weeks, so it can be assumed that the activities of ^226^Ra and ^214^Bi are equal at the time of the measurement. Although we are not aware of any experimental evidence that ^222^Rn would escape from the solid samples, it is useful practice to hermetically seal the samples in small containers.

For determining ^214^Bi activity, the intensity of the 609.3 keV gamma line (and some other ^214^Bi lines) can be recorded by a large coaxial HPGe spectrometer under low-background conditions. The same spectrum is used to determine ^238^U peaks for relative efficiency. As ^214^Bi is a cascade emitter, true coincidence summing losses can cause a bias for short sample-to-detector distances, and should preferably be corrected for. However, for larger sample-to-detector distances the bias due to true coincidence summing losses can often be neglected compared to other sources of uncertainty.

The line of ^214^Pb at 352 keV does not suffer from true coincidence summing losses. However, it is usually difficult to quantify due to its low intensity and high background continuum. Furthermore, it is far away from the peaks which are used to construct the relative efficiency curve, so the uncertainty of the relative efficiency at 352 keV is very high.

The ^226^Ra/^238^U activity ratio determined through measuring ^226^Ra descendants is divided by the ^234^U/^238^U ratio obtained during the measurement of the U isotopic composition. The age of the sample is then calculated from the ^226^Ra/^234^U ratio. ^234^U is preferentially enriched along with ^235^U in the enrichment process. Hence, for lower ^235^U abundances, the amount of ^234^U (and therefore of ^214^Bi) is lower as well, so the corresponding activity is more difficult to measure.

**Fig. 2:**
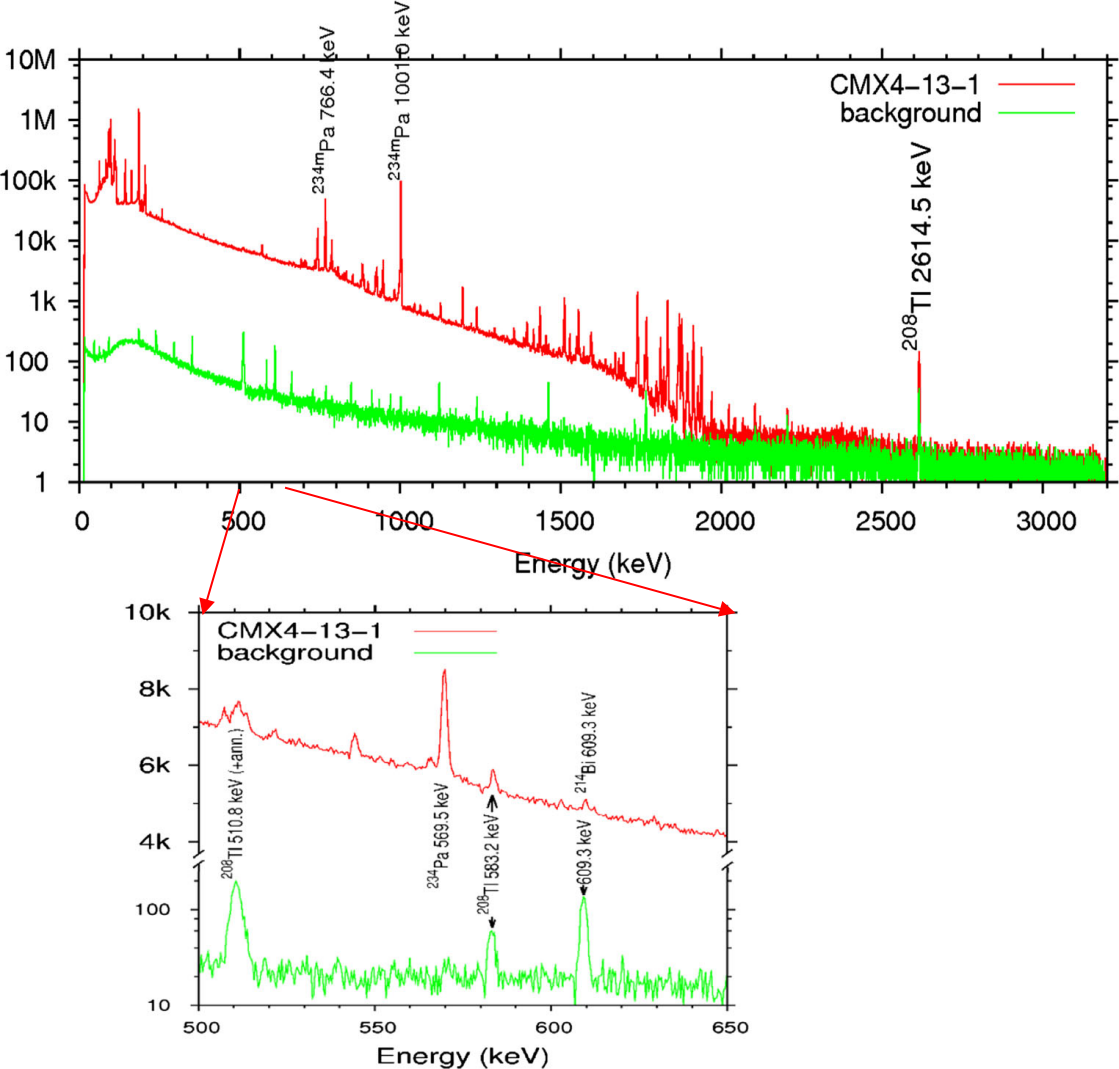
Spectrum of ES-3 (live time = 55,008 s) and of the background (live time = 65,122 s) taken by a well-type HPGe detector in a low-background iron chamber. Both spectra are normalized to 60,000 s live time (Picasso). No surplus of the peak area of ^214^Bi at 609 keV was observed above background. Abundance of ^232^U was evaluated from the net peak areas at 583, 860, and 2614 keV of ^208^Tl, and those at 238 and 727 keV of ^212^Pb and ^212^Bi, respectively

To extend the capabilities of the method, a high-efficiency, 293 cm^3^ well-type detector was acquired by a participant laboratory (Picasso). The first application of this kind of detector for uranium age dating was assaying the CMX-4 exercise samples [20]. The well-type HPGe detector (Canberra GCW6023) was in an iron chamber of 20 cm wall thickness. A spectrum of ES-3 acquired by the well-detector is shown in Fig. 2. Owing to their low enrichment and age, an upper limit of ≈ 11 years was estimated uniformly for the three samples. This result was consistent with the results from destructive measurements.

Ten participant laboratories employed mass spectrometry and alpha spectrometry for determining ^234^U and ^230^Th in the samples [21]. One of those laboratories applied ^231^Pa/^235^U chronometer as well. Most of the measured ages (around 10 years for ES-1 and ES-3 and around 12 years for ES-2) were consistent with the known history of the material.

### Identification of reprocessed material

Gamma spectroscopy is useful for screening uranium samples for fission products, and some of the actinide isotopes that would be produced by neutron activation. If these were detected, they would indicate reprocessed uranium, and also offer clues about the reprocessing technology by identifying deficiencies in the uranium purification chemistry. No fission or activation products were detected in the CMX-4 samples, and upper limits reported for representative nuclides were similar between the three samples.

The minor isotopes ^236^U and ^232^U are characteristic of reprocessed material (or blended/contaminated with reprocessed material) and their presence gives evidence of previous neutron irradiation (e.g., in a reactor) of the sample. The very low ^232^U concentration can be measured by alpha- [22, 23] or gamma spectrometry [18, 24–26].

Some participants of CMX-4 detected ^232^U by HRGS, using heavy shielding for low-background counting:

- Gauguin reported (5.6 ± 3.8) × 10^−11^% ^232^U concentration in ES-1, whereas the two pellets were given upper limits as 8.8 × 10^−11^ and 6.3 × 10^−11^%.
- Vermeer identified 238, 583, and 727 keV gamma lines of ^212^Pb, ^208^Tl, and ^212^Bi, respectively, (descendants of ^232^Th and ^232^U alike) in ES-1 and ES-3 samples, using a low-background system (15 + 5 cm Pb; the inner 5 cm layer is of a low ^210^Pb source), but the 911 keV line of ^228^Ac (daughter of only ^232^Th) was missing. Hence, it was concluded that the former did not derive from ^232^Th, but rather from ^232^U (decay scheme of the two nuclides is common starting from ^228^Th). However, they could not find any of those lines in the ES-2 pellet.
- Picasso evaluated ^208^Tl, ^212^Bi, and ^212^Pb peaks in spectra of ES-1 and ES-3 samples, in absence of ^228^Ac, but no such peaks for ES-2 above background. The 2614 keV ^208^Tl peak was observed in the spectrum of ES-1 (also ES-3, Fig. 2) sample, corresponding to (6 ± 0.5)10^−11^% ^232^U concentration, but difficult to exclude from background (≈ 10^−11^%) in the ES-2 sample. From the lack of the 911 keV line of ^228^Ac, Picasso estimated a detection limit of about 10^−4^ for the ^232^Th/^238^U mass ratio with a 12 h measurement time using the well detector.
- Buonarroti performed analysis similar to that of Picasso, and obtained that ES-1 and ES-3 contain (1.40 ± 0.96)10^−10^% and (1.20 ± 0.70)10^−10^% of ^232^U, respectively, whereas ES-2 contains less than 1 × 10^−10^%.

Presence of ^232^U in ES-1 and ES-3 indicates that the samples were manufactured from material mixed or contaminated with reprocessed uranium. According to mass spectrometric results [1], the concentration of ^236^U in both samples is about 0.0020 ± 0.0004% on average, but near the detection limit in ES-2 (≤ 10^−4^). This confirms the conclusion from HRGS that ES-1 and ES-2 contained some reprocessed material, while ES-2 did not.

^236^U cannot be analyzed in LEU by gamma spectrometry, only in extremely high-enriched (weapons-grade) material [13], because its peaks are masked by the much stronger peaks from the major U isotopes (e.g., the strongest, but still quite weak 49.369 keV line of ^236^U lies very near the 49.55 keV line of ^238^U.) Thus, ^236^U abundances were only quantified by mass spectrometry.

A correlation between the ^236^U and ^232^U contents exists. According to Picasso’s results, the ^236^U/^232^U abundance ratio was ≈ 3 × 10^7^ in the ES-1 and ES-3 samples. This value is in agreement with earlier results [18, 24, 26] on U samples over the full range of enrichments, from LEU to the highest enriched (90%) uranium, and is in accordance with theoretical predictions [27].

It is not clear where the reprocessed U in samples ES-1 and ES-3 comes from. All the three samples were made in the same factory (fictitious “EA Fuel Products” or virtually HIFAR [1]) and from natural U. If they got contaminated with reprocessed U in the enrichment plant, all three should contain traces of ^236^U and ^232^U, unless the facility became contaminated between processing the two source materials A and B.

## Conclusions

For identifying the provenance of unknown nuclear material, information on the isotopic composition, the age, and previous neutron irradiation of the material is relevant. In addition to previous exercises when weapon grade materials were examined, this exercise confirmed that gamma spectrometry also plays a significant role for the analysis of LEU in the comprehensive response to these issues.

Results of this exercise confirmed that LEU can be categorized as such via gamma spectrometry within 24 h, regardless of the detector, software, and calibration methodology. For accurate determination of isotope ratios, the best results were acquired with planar HPGe detectors and current versions of the MGAU and U235 software. The utility of high-efficiency HPGe detectors in low-background setup was demonstrated for detecting trace ^232^U, thus indicating contamination with reprocessed U. Challenges related to the age dating of low-enriched and young aged uranium (difficulty with determination of lower amounts of ^214^Bi) were identified.

The combination of different analytical techniques increases the confidence in the results and can help further narrow down the set of possible origins and intended uses of the examined materials.
